# The Functional Test for Agility Performance is a Reliable Quick Decision-Making Test for Skilled Water Polo Players

**DOI:** 10.1515/hukin-2015-0044

**Published:** 2015-07-10

**Authors:** Guilherme Tucher, Flávio Antônio de Souza Castro, António José Rocha Martins da Silva, Nuno Domingos Garrido

**Affiliations:** 1 University of Trás-os-Montes and Alto Douro, Vila Real, Portugal. Research Centre in Sports Sciences, Health Sciences and Human Development, CIDESD, Portugal.; 2 Federal University of Rio Grande do Sul, Porto Alegre, Rio Grande do Sul, Brazil.

**Keywords:** athletic performance, training, functional performance, assessment, aquatic sports

## Abstract

The reliability of the Functional Test for Agility Performance has only been evaluated in water polo players in a small group of novice athletes. Thus, the aim of this study was to evaluate the reliability of the Functional Test for Agility Performance in skilled water polo players. Forty-two athletes (17.81 ± 3.24 years old) with a minimum of 5 years of competitive experience (7.05 ± 2.84 years) and playing at the national or international level were evaluated. The Functional Test for Agility Performance is characterized as a specific open decision-making test where a tested player moves as quickly as possible in accordance to a pass made by another player. The time spent in the test was measured by two experienced coaches. Descriptive statistics, repeated measures analysis of variance (ANOVA), 95% limit of agreement (LOA), intraclass correlation coefficient (ICC) and standard error of measurements (SEM) were used for data analysis. Athletes completed the Functional Test for Agility Performance in 4.15 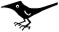 0.47 s. The ICC value was 0.87 (95% IC = 0.80–0.92). The SEM varied between 0.24 and 0.38 s. The LOA was 1.20 s and the CV average considering each individual trial was 6%. The Functional Test for Agility Performance was shown to be a reliable quick decision-making test for skilled water polo players.

## Introduction

In team sports, an athlete’s speed and agility are some of the most important motor capacities assessed (Sheppard and Young, 2006; Lockie et al., 2013). However, it is important to know the specificity of the test used for evaluation of these capabilities (Uljevic et al., 2014). In water polo studies, researchers attempt to evaluate the athlete’s performance in water during tasks with similar duration as those actions which occur in the game (Mujika et al., 2006; Tan et al., 2010; Uljevic et al., 2014). However, these proposed tests are concerned only with conditioning capacities, disregarding the importance of perceptual skills and decision-making. Therefore, this method of testing can be considered to be similar to closed skill tests. On the other hand, in team sports, athletes are frequently required to make decisions quickly regarding their actions. Therefore, real situations – specific necessity, in team sports are considered open skill tasks (Falk et al., 2004; Jackson et al., 2006; Sheppard and Young, 2006).

From recent studies in team sports, the importance of decision-making and anticipation skills to perform technical and tactical tasks correctly and to differentiate players based on skill levels has become evident (Falk et al., 2004; Moir et al., 2004; Sheppard and Young, 2006). Uljevic et al. (2014) recently found that combined-capacity tests discriminated qualitative groups of junior water polo players more effectively than single-capacity tests. However, the authors were still concerned only with physiological capacities. Furthermore, as the literature highlights (Lupo et al., 2010; Lupo et al., 2011), teams which are technically and tactically well-matched perform more actions next to the goal in the vertical position, in various directions and different planes. To assess vertical and directional movement in accordance with decision making (passing), only one study has been published (Tucher et al., 2014). However, in this preliminary study, the reliability was evaluated using only fifteen novice water polo players. Thus, believing that Functional Test for Agility Performance (FTAP) is an important test that should be used either with more experienced water polo players, that reliability studies should test an adequate sample size (Hopkins, 2000; Shoukri et al., 2004), and that reliability is dependent on the design of the study and the population (Beckerman et al., 2001), it is relevant to assess the reliability of the FTAP with skilled water polo players. According to the FTAP description, we hypothesized that it should be an ideal test to assess defensive actions.

In water polo perceptive and cognitive capabilities are important to performance. During situations near the goal, the defensive players have to pay attention to the ball, passes and opponent actions (Escalante et al., 2011; Lupo et al., 2012a; Lupo et al., 2014). It is noteworthy because offensive players are trying to pass the ball speedily to outmanoeuvre defensive players (Lupo et al., 2010). Thus, defensive players should have great perceptual capacities because they need to work well in even and power play situations (Lupo et al., 2010; Lupo et al., 2012a). Lupo et al. (2012a), for instance, showed that winning teams perform fewer even offensive actions and more counterattacks than losing teams. One reason is because defensive players can stop the opposite offensive action by stealing the ball. Even this, defensive players and goalkeeper actions were one of the most important factors between men winning teams in the Olympic Games of 2008 (Escalante et al., 2011).

Therefore, the aim of this study was to assess the reliability of the FTAP in skilled water polo players in accordance with studies in the literature (Hopkins, 2000; Falk et al., 2004; Impellizzeri and Marcora, 2009). The total time measured in the FTAP test is a result of the speed of decision-making, the technique used for changes of direction and the swimming skills used for moving several meters. Our hypothesis was that FTAP reliability would be confirmed. This may facilitate the selection process, provide information about the actual physical condition of an athlete and may also be used for measuring the effectiveness of training in specific quick decision-making performance tests.

## Material and Methods

### Participants

The Federal University of Rio Grande do Sul Ethics Committee approved the methods and procedures (70263/2012) and the study was conducted in accordance with the Declaration of Helsinki. Forty-two male water polo players (37 perimeter players, 3 center players and 2 goalkeepers; 17.8 ± 3.2 years of age; 178.3 ± 7.2 cm of body height) with at least 5 years of water polo training (7.0 ± 2.8 years), volunteered for this study. The players were at the national or international level and were involved in 5.8 ± 1.7 training sessions per week (120 min each). This sample size is in accordance with literature recommendations (Hopkins, 2000; Shoukri et al., 2004).

### Procedures

All athletes and evaluators were instructed together regarding the test procedures and performed five FTAP familiarization trials. The familiarization trials are important to (1) athletes being tested, (2) to the players responsible for the passes and (3) to evaluators. The evaluators were the same in all testing. Any queries were addressed ensuring that all participants understood the procedures before testing. The FTAP was performed between 6.30 pm and 8.30 pm, one day after the familiarization session. Participants were instructed to refrain from exercise in the morning of the testing day. There was a standardized warm-up that consisted of dry-land stretching and dynamic joint mobility exercises, 200 m free-swim alternating front and back strokes and various kick styles, 4 × 100 m front-crawl swims with no-push turns every 25 m, starting every 110 s, and 4 × 25 m (12.5 m sprint, 12.5 m recovery), starting every 50 s.

The total time to perform the test was manually measured in seconds using two sport chronometers (Professional Stopwatch Vollo Concept – model VL233, P. B. Yang Sport, China) by two experienced coaches, named evaluators A and B. The evaluator began to record the testing time from the moment when the tested player removed his hand from the ball (Picture 1A–1B). Timing was stopped when the tested player removed the second ball from the arch (Picture 1H), defining the total time for the test. The final time obtained by each evaluator for each athlete was recorded after the end of the test.

To evaluate the agility of the players in a quick decision-making test, the FTAP was used as previously described (Tucher et al., 2014). In the FTAP, the athlete should move as quickly as possible in a 3 m square in accordance to the random pass made by another player. The tested player was within the FTAP square (in one of its extremities) and had one hand on an official water polo ball that was floating in one arch nearby. This was considered the start (Picture 1A, tested athlete was touching the ball). Another four players were positioned outside each of the four FTAP square corners with one ball in each arch (Picture 1). The player next to the tested athlete had a fifth ball in his hand (Picture 1A). When this player perceived that the tested player had removed his hand from the floating ball and begun a fast movement towards the center of the square, he threw the ball immediately to the player in the opposite direction (this movement was obligatory) (Pictures 1B and 1C). Upon receiving the ball, this player then passed the ball to one of the players at his side (his right or left) (Pictures 1C, 1D and 1E) without indicating the intended direction in order to avoid any anticipation from the tested player. When this movement occurred, the player being tested should move as quickly as possible to where the ball had been passed and remove the ball floating in the arch using any part of his body (Pictures 1E, 1F and 1G). The player who received the ball should then pass it once again (his right or left) (Pictures 1E, 1F and 1G). Again, the tested player removed the ball floating in the arch using any part of his body (Picture 1H). The test was then completed.

The tested player did not know in advance to whom the ball would be passed. In addition, the four other players and the destination of the passes were randomly chosen between trials by the athlete responsible for the pass. The tests were repeated three times for each individual and a minimum of three minutes of rest was allowed between trials. If any unexpected factor occurred that could hinder the performance of the test (tested player moved inappropriately or errors in pass, for example), the procedure for the same tested player was performed after the next athlete in line had been tested.

### Statistical Analysis

The normality of all measurements was assessed using the Kolmogorov-Smirnov test. The mean, standard deviation (SD), and coefficient of variance (CV) were calculated for within-trials and between-trials. The Mauchly test was used to test the sphericity assumption for the evaluator effect, the trial and the evaluator vs. trial interaction. An ANOVA for repeated measures in a mixed 3 by 2 model (trial vs. evaluator) with Bonferroni’s post hoc was used to test the influence of factors (evaluator, trial and interaction between evaluator and trial) on the results. The test of within-athletes contrasts was used to compare the independent variable. Effect sizes were obtained by contrasts result, where F value was converted to r. An r = 0.50 represented a large effect size (Field, 2009). In all cases, α ≤ 0.05 was defined for significant differences and/or interactions.

The 95% limit of agreement (LOA) was calculated by summing up the difference mean from evaluators A and B (d) with a product of ± 1.96 by the SD of the difference between the mean of evaluators A and B (s_d_) (thus, LOA = d ± 1.96^*^SD). The procedure used to calculate the intraclass correlation coefficient (ICC) was the two-way random model of the absolute agreement. The ICC for mean measures was considered since each athlete was assessed three times by two independent evaluators (six results for each athlete). The standard error of measurements (SEM) was obtained by the square root of the mean quadratic error from the twoway ANOVA for repeated measurements (Eliasziw et al., 1994; Atkinson and Nevill, 1998). All statistical modeling was performed using a statistical package (IBM-SPSS, v. 20, Chicago, USA).

## Results

Including the three trials for each evaluator, the forty-two athletes completed the FTAP in 4.15 ± 0.47 s (CV of 11.0%) (n = 252 trials). For all trials of evaluator A, the results of the FTAP were 4.13 ± 0.23 s (CV = 6%) and for evaluator B, the values were 4.17 ± 0.24 s (CV = 6%). The mean values for each individual trial and each evaluator are shown in Table 1.

All data assumed normal distribution (p > 0.05). The hypothesis of sphericity in distribution was confirmed for the effect of trial (x^2^(2) = 0.63, p = 0.72) and interaction trial-evaluator (x^2^(2) = 0.013, p = 0.99). No differences were found within-athletes for (1) each trial (F(2, 82) = 0.68, p = 0.50); for (2) evaluator (F(1, 41) = 0.63, p = 0.43); or for (3) the interaction trial-evaluator (F(2, 82) = 1.29; p = 0.28). The test of within-athletes contrasts showed trial 1 (F(1, 41) = 1.27, r = 0.17) and trial 2 (F(1, 41) = 0.91, r = 0,14) were not significantly higher compared to trial 3. The same was found between evaluators (F(1, 41) = 0,63, r = 0.10) and trial-evaluator interaction when trial 1 and trial 3 for both evaluator (F(1, 41) = 0.94, r = 0.15), and trial 2 and trial 3 for both evaluator (F(1, 41) = 2.50, r = 0.24) were considered. In all cases the effect sizes were considered small (Field, 2009).

**Figure 1:**
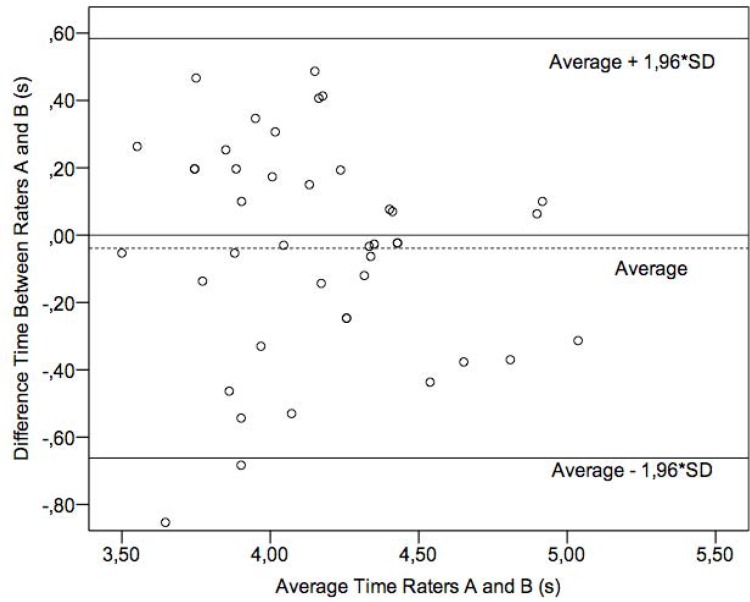
FTAP performance time: difference of time (evaluator A minus evaluator B) versus average time measured by evaluators A and B with the 95% limit of agreement (SD = standard deviation).

The mean difference between evaluator A and evaluator B was − 0.04 ± 0.31 s. The difference presented a normal distribution (p = 0.16). Therefore, it could be expected that, in 95% of the cases, the difference between measurements registered by the evaluators would be between −0.64 s and 0.56 s (range of 1.20 s), which characterizes the 95% limit of agreement (LOA) (Figure 1). These values represent an amplitude for the value obtained of 1.20 s (Bland and Altman, 1999). The ICC was 0.87 (95% IC = 0.80–0.92) for p < 0.01. The SEM found for the trial effect was 0.35 s, for the evaluator was 0.38 s and for the interaction trial-evaluator was 0.24 s.

## Discussion

The aim of the present study was to assess the reliability of the FTAP in skilled water polo players in accordance with previously described methods in the literature. To our knowledge, the present study is the first to assess the reliability of a quick decision-making test in skilled water polo players. Although there are tests to evaluate water polo players, they do not represent open skill tests linked to team sports performance. In the FTAP the athlete should move as quickly as possible in a 3 m square in accordance to the random pass made by another player. Our hypothesis was confirmed and the FTAP showed high reliability in this group of skilled water polo players assessed as described in the literature (Hopkins, 2000; Falk et al., 2004; Impellizzeri and Marcora, 2009). Furthermore, the short and fast swimming movements of defensive players are crucial, for instance, to face the high offensive impact of the opponent center forward during even action (Lupo et al., 2012b), as well as during defensive arrangements occurring during power-play (Lupo et al., 2012a; Lupo et al., 2014).

No significant differences among any of the three trials or evaluators were found in the present study. This result indicates there was no learning effect regarding those measures. The FTAP contains some characteristics of unpredictability, which may increase variability, but results showed appropriate values of ICC – near other tests with closed skill characteristics (Moir et al., 2004; Uljevic et al., 2013). In the same manner, despite the acceptable CV (≅ 6%) (Atkinson and Nevill, 1998), others studies showed lower values for measurement error. One study found values of the CV near 1.9–2.6% (Moir et al., 2004) while another near 2.0–9.0% (Uljevic et al., 2013). The higher CV observed for the FTAP in the present study is likely to be due to the shorter duration of the test (Hopkins et al., 2001) and to the open skill characteristics used in the study (i.e. increasing variability). In a previous study (Lockie et al., 2013), the reliability of 20 m sprint tests (0–5, 0–10, 0–20 m intervals) was evaluated. It was shown that the shorter the distance, the greater the CV (5.1% for 0–5m vs. 1.9% for 0–20 m). It has been proposed that power tests of longer duration have lower within-athlete variation because the random error associated with variance in limb movements is cancelled by the larger numbers of repetitions (Hopkins et al., 2001). Moreover, it is important to indicate that movements in the water are different compared to those on land. As the drag in water is high, any wrong interpretation of the scene by the athlete could impair the next movement (Jackson et al., 2006; Uljevic et al., 2013).

The number of familiarization trials was higher than that used in a preliminary FTAP study (5 vs. 2) (Tucher et al., 2014). A greater number of familiarization trials were conducted to avoid potential learning effects and to decrease variability. The ICC results in the present study support the importance of familiarization trials in the FTAP as they allow athletes to practice various possibilities for movements that can be employed. Some authors (Moir et al., 2004) evaluated the influence of familiarization on the reliability of the vertical jump and acceleration sprinting (running) performance in physically active men. They concluded that reliability could be assessed without the need to perform familiarization sessions. It is important to state that the vertical jump and sprint running are familiar tasks to healthy active individuals. Even in some water polo tests, familiarization may not be necessary (Uljevic et al., 2013; Uljevic et al., 2014). Therefore, motor skills required, experience of the athlete and the nature of the test should be considered to evaluate the need for familiarization trials (Moir et al., 2004). However, it is emphasized that familiarization is important in tests such as the FTAP, where perceptual and decision-making tasks are important.

In the present study more athletes were evaluated compared to the other study conducted to evaluate FTAP reliability (Tucher et al., 2014) and other studies evaluating reliability in sports tests (Rechichi et al., 2000; Mujika et al., 2006; Platanou, 2006). In addition to the increased number of assessed athletes, our investigation evaluated skilled male water polo players. This is important because reliability is dependent on the design of the research and the population being studied (Beckerman et al., 2001). In comparison to the first FTAP study by Tucher et al. (2014), which involved players with a minimum of 2 years of experience and with different skill levels, lower mean time to complete the FTAP was found (4.15 s vs. 4.73 s) and close the CV (6%). It is because better athletes achieved higher scores of decision-making during water polo games by using subjective coaching analysis as a reference (i.e. anticipating on-going activities and making appropriate decisions) (Falk et al., 2004). Furthermore, we believe that skilled water polo players move significantly faster and spend less time making decisions compared to novice players (Veale et al., 2010; Young and Willey, 2010).

However, in the present study, values of the LOA and SEM were higher. Although for trial effect, the SEM was lower. This represents greater consistency in the performance of skilled athletes compared with novices. Although no significant differences between-evaluators were observed, the LOA equaled 1.20 s and SEM was 0.20–0.40 s (for the trial effect it was 0.35 s, for the evaluator it was 0.38 s and for interaction trial-evaluator it was 0.24 s). Considering the presented values of the LOA and SEM, it is proposed that the mean time of the three trials performed by an athlete can be considered as the reference of performance in the FTAP. This mean value should be used in training for comparison of performance (between and within-athletes) or in comparison to other studies.

## Conclusions

The FTAP was shown to be a reliable quick decision-making agility test for skilled water polo players and can be considered a coherent test with respect to water polo game situations. For optimal reliability of assessment it is important to reduce measurement error with special attention to familiarization of athletes and training of the evaluators in the test. Water polo athletes are generally assessed in closed skill tests but emphasis should be placed on evaluation of perception and decision-making together with physiological capabilities which occur in real game situations. This occurred in the FTAP test because the total time measured is a result of the speed of decision-making, the techniques used for change of direction and the swimming skills for moving several meters in water. Finally, the FTAP was shown as an important test to monitor water polo players and should be considered by coaches and physical trainers to evaluate specific game aspects like decision-making and swimming abilities for changes of direction.

## Figures and Tables

**Picture 1:**
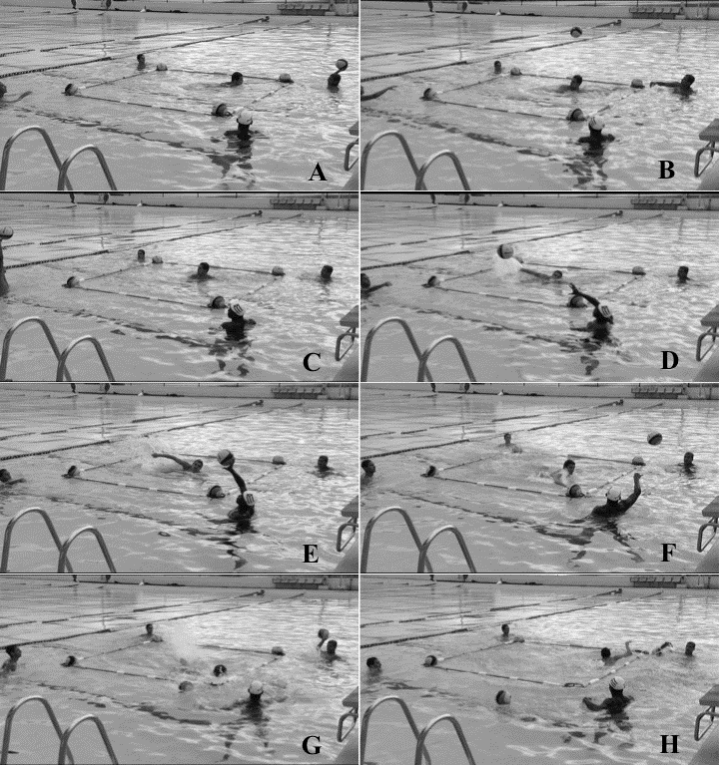
Functional Test for Agility Performance (FTAP) to evaluate water polo players. Picture 1A. Start of the test - the player being tested is within the FTAP square and has one hand on a ball. Picture 1B–1C. First pass – the tested player moves to the center of the square. Picture 1D–1E. Second pass – the tested player moves where the ball has been passed and removes a ball that is floating in the arch. Picture 1F–1G. Third pass – the tested player moves where the ball has been passed again and removes a ball that is floating in the arch. The test is then completed.

**Table 1:** Results of the FTAP presented as descriptive measurements for each individual trial and each evaluator.

	Evaluator A			Evaluator B		

	Mean (s)	SD (s)	CV (%)	Mean (s)	SD (s)	CV (%)
Trial 1	4.15	0.51	12.0	4.20	0.49	12.0
Trial 2	4.12	0.35	8.0	4.21	0.52	12.0
Trial 3	4.13	0.44	11.0	4.10	0.50	12.0
Overall	4.13	0.43	10.3	4.17	0.50	12.0

Mean, standard deviation (SD) and coefficient of variation (CV).
